# Amiodarone-induced Hemoptysis: A Rare Presentation of Amiodarone-induced Pulmonary Toxicity Occurs at a Low Dose

**DOI:** 10.7759/cureus.5289

**Published:** 2019-07-31

**Authors:** Clayton D Busch, Caleb J Heiberger, Tej I Mehta, Douglas Yim

**Affiliations:** 1 Anesthesiology, University of South Dakota, Sanford School of Medicine, Sioux Falls, USA; 2 Radiology, University of South Dakota Sanford School of Medicine, Sioux Falls, USA; 3 Interventional Radiology, Avera McKennan Hospital and University Health Center, Sioux Falls, USA

**Keywords:** amiodarone induced pulmonary toxicity, diffuse alveolar hemorrhage, hemoptysis, hypoxemic respiratory failure

## Abstract

Amiodarone-induced pulmonary toxicity (APT) is one of the most feared and underappreciated adverse effects of this commonly prescribed antiarrhythmic. APT has a variable presentation, among the rarest of these is amiodarone-induced diffuse alveolar hemorrhage with hemoptysis. Though previous cases confirmed with biopsy averaged a dose of 570 mg PO daily, APT can occur at any dose. Previous literature has suggested the importance of cumulative exposure to amiodarone rather than the patient’s actual dose. The presented case describes amiodarone-induced hemoptysis occurring at a dose of 200 mg PO daily for five years. Additionally described is the treatment regimen which managed a patient with metabolic syndrome and elevated A1c while addressing the recommended treatment of extended high-dose steroids for APT with complicated respiratory status. To the best of the authors’ knowledge, only two biopsied cases have been described at a dose this low. Furthermore, this case describes a more typical timeline for APT than those two cases.

## Introduction

Amiodarone, one of the most frequently prescribed medications in the U.S., can cause amiodarone pulmonary toxicity (APT). APT is a potentially fatal and underrecognized drug complication [1, 2]. APT presenting with diffuse alveolar hemorrhage (DAH) and hemoptysis is a rare occurrence with only 10 biopsy proven events as of 2006 [3]. Borders et al. identified 15 cases as of 2012 regardless of biopsy status, and an additional case was described in 2018 [4, 5]. On average, biopsied patients had been on amiodarone for 5.6 months at a dose of 570 mg prior to experience alveolar hemorrhage and hemoptysis symptoms, and two of these cases reported at a dose of 200 mg which had been given for 14 and 200 days, respectively [3]. In comparison, Martin and Rosenow describe APT of any kind occurring in 5-7% of patients taking amiodarone, including doses as low as 200 mg, but these typically occur after “years of therapy” [6]. This case reports a patient on 200 mg of amiodarone for five years to treat paroxysmal atrial fibrillation that experienced DAH with hemoptysis.

## Case presentation

A 73-year-old female with past medical history of hypothyroidism, class III obesity, myocardial infarction with four vessel coronary artery bypass grafting (CABG), and paroxysmal atrial fibrillation controlled with 200 mg amiodarone for five years presented to an outlying clinic with “vision trouble”. Symptoms began in one eye that morning and progressed to bilateral involvement over a few hours. Laboratory findings were significant for an erythrocyte sedimentation rate (ESR) of 115. Optometry diagnosed her with bilateral retinal artery occlusion. High dose IV steroids were promptly initiated for coverage of giant cell arteritis (GCA). She was admitted for further workup with temporal artery biopsy and echocardiogram. A carotid ultrasound, magnetic resonance imaging (MRI), and magnetic resonance angiogram (MRA) were also performed to evaluate for possible embolic etiology. The echocardiogram showed atrial fibrillation with moderate aortic stenosis and mild-moderate mitral stenosis. Carotid ultrasound showed extensive atherosclerosis without evidence of complete occlusion. MRI and MRA were unremarkable; temporal artery biopsy results were negative for any acute pathology.

**Figure 1:**
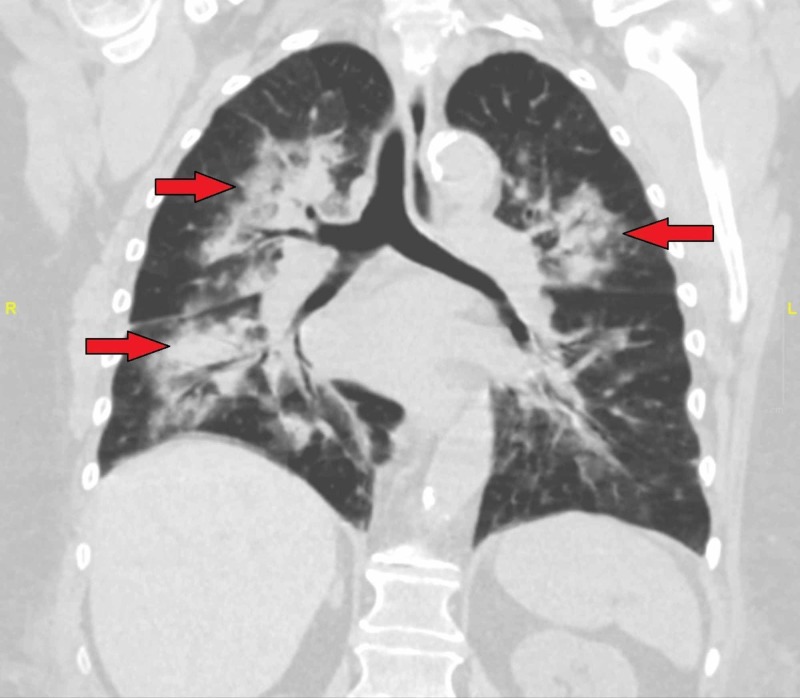
Post-hemoptysis CT of chest Red arrows indicating bilateral opacities appreciated on CT scan consistent with diffuse alveolar hemorrhage vs. pneumonia. Diffuse alveolar hemorrhage was subsequently confirmed on bronchoalveolar lavage.

Four days after her initial presentation, the patient suddenly became hypoxemic with a cough productive of bloody expectorant. Seven liters (FiO2 of 44%) of supplemental oxygen were necessary to maintain saturations over 90%. A CT of her chest was performed, and it demonstrated extensive patchy bilateral areas of airspace opacity and consolidations and an enlarged heart with no interval change in heart size (Figure 1). These findings were suggested by radiology to be secondary to pneumonia vs. pulmonary hemorrhage. Empiric ceftriaxone and azithromycin were both started at this time. Amiodarone and warfarin therapies were stopped, but no reversal agents were given for warfarin since her international normalised ratio (INR) was 1.09 at that time. Baseline hemoglobin levels ranged from 10.7 to 11.5 g/dL (normal 12-16 g/dL), but hemoglobin in the acute setting was measured at 9.9 g/dL. She was promptly transferred to a tertiary care center ICU.

The following day in the ICU further workup was performed with a bronchoalveolar lavage (BAL) demonstrating frank blood in three consecutive aspirates consistent with DAH. Furthermore, a thick layer of blood was observed along the bronchi without evidence of laceration, mass, or damage that could be attributable to the pulmonary bleeding that appeared worse in more distal segments of the lungs where collections of blood were appreciated. ESR remained elevated above 80 mm/hr (normal 0-29 mm/hr) throughout her hospital stay despite a constant regimen of high dose steroids. White blood cells (WBC) remained elevated throughout most of her symptomatic period with a peak of 13.0 cells/mcL (normal 4.5-11.0 cells/mcL) eight days after her initial presentation. BAL samples were sent for microbiological testing with all results coming back negative including fungal cultures, respiratory syncytial virus (RSV), legionella, acid fast bacilli smear, and gram stain. Sputum sample and peripheral venous blood also produced negative gram stain and final cultures. An extensive rheumatologic workup was negative including antinuclear antibody (ANA), antineutrophil cytoplasmic antibody (ANCA), and anti-glomerular basement membrane (anti-GBM). Additionally, ultrasound of both legs was performed showing no evidence of deep vein thrombosis (DVT). Brain natriuretic peptide (BNP) was recorded at 672 (normal 0-100 pg/mL) which was elevated from her baseline of approximately 300 pg/mL. PT/INR was also unremarkable with constant values below 2 for the duration of her visit. Complete blood count (CBC) with differential was normal beyond the abnormalities previously described. Eosinophils were also within the normal reference range (0-0.45 cells x 109/L). Hemoglobin gradually dropped over the next week to a minimum of 8.1 g/dL at which time she received a transfusion. Her oxygen needs also increased along this timeframe.

During her ICU stay oxygen needs increased to a peak of 65% FiO2 via mask delivery. Her oxygen needs diminished gradually, and she was transferred to a rehab unit adjacent to the ICU. In the rehab unit the patient was able to ambulate and function safely and independently on her decreasing supplemental oxygen needs. Fifteen days after her initial presentation she was discharged with stable vital signs on room air. There was no recurrence of dyspnea or related symptoms at three-month follow-up.

## Discussion

Amiodarone is an efficacious antiarrhythmic with activity against most arrhythmias, both ventricular and supraventricular [1]. It also carries a spectrum of side effects including corneal deposits, discoloration of skin, bone marrow suppression, and peripheral neuropathies. Its most severe side effect is APT [6, 7]. Meta-analysis suggests the rate of APT incidence is 1% per year on amiodarone [8]. Another source suggests approximately 5-7% of patients taking amiodarone will get APT and in 5-10% of these patients it will be fatal [6]. Risk factors for lung complications with amiodarone use include older age, duration of treatment, cumulative dose of medication and its metabolites, history of cardiothoracic surgery, high oxygen use, use of contrast, co-existing respiratory infections and pre-existing lung pathology [9]. Presentation of APT is non-specific, but it is typically classified as either acute or insidious onset. Acute onset is described in one-third of patients with APT, occurring after a couple weeks on amiodarone. These patients present with fever and an acute pneumonitis a couple weeks after taking amiodarone. Heart failure symptoms are also possible. The insidious onset is associated with at least two months of therapy and is typically characterized by progressive nonproductive cough, dyspnea, weight loss, and sometimes fever with interstitial infiltrates on chest x-ray [6].

It is important to note that toxicity is better correlated with total cumulative dose than either the patient’s daily dose or plasma level of amiodarone [7]. Toxicity can occur at any time, but Wolkove and Baltzan describe typical timelines as anticipated by dose: patients taking at least 400 mg for more than two months and patients taking 200 mg or less for more than two years. Oral amiodarone specifically has been observed to accumulate dramatically in lung and adipose tissue [7]. With class III obesity and a low dose of oral amiodarone, one would expect a later presentation of APT. As both lungs and adipocytes act as reservoirs for the drug, a larger adipocyte reservoir competing for accumulation could delay the onset of pathology in the lungs.

Diagnosis of APT should include three or more of the following: new or worsening dyspnea or new findings on lung exam, abnormalities on imaging, abnormalities in total lung capacity or diffusion lung capacity (DLCO), CD8+ lymphocytes in BAL fluid, lung biopsy showing DAH, interstitial pneumonitis, pulmonary fibrosis or organizing pneumonia [10]. The presented case met diagnostic criteria as the patient had new dyspnea, bilateral patchy opacifications on chest CT, and diffuse crackles appreciated throughout all lung fields on physical exam. Further evaluation of the case using the Naranjo Adverse Effect Probability Scale indicated the diagnosis was probable [11].

DLCO was not measured, but likely would have been altered as the carbon monoxide used to evaluate DLCO is sequestered by the blood occupying the lungs [12]. Furthermore, the clinical instability of our patient made performing DLCO testing impractical. Similar logic supported the decision to forego lung biopsy. Despite biopsy being the gold standard of diagnosis, it is rarely indicated as APT worsens with cardiothoracic surgery and patients typically are not optimal surgical candidates owing to their cardiopulmonary issues [7].

Important considerations for differential diagnosis in patients with APT with hemoptysis include pulmonary embolism, malignancy, pneumonia, eosinophilic pneumonitis, acute congestive heart failure (CHF), autoimmune disease, and warfarin-induced hemoptysis [3, 6]. The presented case did have unilateral leg edema, but she described this as her baseline since her saphenous vein was harvested for her CABG. Additionally, ultrasound of the lower-extremities showed no evidence of thrombosis. CT and BAL failed to demonstrate evidence of malignancy. BAL, sputum sample, and peripheral venous blood all came back negative for infection. CBC with differential demonstrated a normal eosinophil count making eosinophilic pneumonitis unlikely. A CHF exacerbation was considered as her BNP was 672 pg/mL with difficulty breathing. She did not, however, have leg edema more pronounced than her baseline, and her weight was also at baseline at the time of hemoptysis. Imaging of her heart showed borderline heart enlargement stable compared to previous radiographs. Given her new onset anemia, increased heart wall stress secondary to pulmonary pathology, and critical status it is reasonable to attribute her transiently elevated BNP to the stress imposed on the heart by her health status. An extensive auto-immune panel also came back negative, including Goodpasture disease.

Warfarin-induced alveolar hemorrhage was also considered. There is a documented interaction with amiodarone inhibiting warfarin processing in the liver through CYP 2C9 and raising risk of pathologic hemorrhage. However, this patient’s INR was 1.09 at the time of hemoptysis making warfarin-induced hemoptysis unlikely as previous case reports emphasize elevated INR values [13, 14]. The current patient had a baseline hemoglobin of 10.7-11.5 g/dL, but it was 9.2 g/dL the day after her presentation with visual symptoms. She did not have hypoxemia or hemoptysis until four days after the initial presentation. Iskandar et al. questioned if subclinical alveolar hemorrhage may be occurring before the acute presentation [3]. The timeline of her hemoglobin dropping before she was started on warfarin and before her hypoxemic hemoptysis suggests warfarin was not the inciting factor and that a subclinical development of DAH was already in progression before clinical symptoms were apparent. Additionally, the patient’s warfarin was restarted by primary care for atrial fibrillation during follow-up. There was no return of pulmonary symptoms.

The most agreed upon therapy for APT is stopping amiodarone. There is less consensus on the use of steroids. Martin and Rosenow recommended two to six months of prednisone 40-60 mg daily. It is left to clinical judgement to decide the risk-benefit analysis of using steroids with respiratory status being a key consideration. Duello et al. elected to avoid steroids entirely as their patient only required a mild oxygen supplement [10]. There were reservations in maintaining such an extensive dose of steroids in a metabolic syndrome patient with questionable A1c. Initially, the patient in the present case report had symptoms of GCA so she was treated with an extensive course of steroids from the outset. Her oxygen needs were monitored inpatient until she was stable on room air both at rest and with activity. Steroids were tapered upon discharge giving her about a month of steroid treatment. This allowed a considerable course of steroids with tapered overlap after she was discharged with a stable respiratory status. Amiodarone was never re-started and no new anti-arrhythmics were started in its place, although her daily medications already included 200 mg of labetalol twice daily. At the point of three-month follow-up there were no complications with her atrial fibrillation management and no recurrence of pulmonary symptoms.

## Conclusions

Hemoptysis is an extremely rare presentation of APT. The current case details a patient receiving 200 mg of amiodarone PO daily for five years. This presentation is similar to previously reported incidences of APT in regards to its presentation after years of therapy, but it is distinguished from the two reports with hemoptysis at 200 mg because those cases had less than a year of amiodarone therapy at their time of hemoptysis. Theoretically, the accumulation of amiodarone in lung tissue could be delayed by another amiodarone reservoir such as adipocytes in a morbidly obese patient. Although lung biopsy is the gold standard for diagnosis, consideration of patient’s health status prior to the workup is vital. Stopping amiodarone in APT-associated DAH is essential. Therapy with steroids should weigh the benefit on respiratory status against the adverse effects on metabolism and other body systems. Recovery of lung status with adequate saturation on room air is a reassuring response to therapy. Close follow-up is advised to monitor for recurrence. This case report described diffuse alveolar hemorrhage in association with amiodarone that was successfully treated by discontinuing amiodarone, giving high dose steroids, and managing respiratory status.

## References

[1] Ashrafian H, Davey P (2001). Is amiodarone an underrecognized cause of acute respiratory failure in the ICU?. Chest.

[2] Sweidan AJ, Singh NK, Dang N, Lam V, Datta J (2016). Amiodarone-induced pulmonary toxicity - A frequently missed complication. Clin Med Insights Case Rep.

[3] Iskandar SB, Abi-Saleh B, Keith RL, Byrd RP Jr, Roy TM (2006). Amiodarone-induced alveolar hemorrhage. South Med J.

[4] Borders CW III, Bennett S, Mount C, Claassen SL (2012). A rare case of acute diffuse alveolar hemorrhage following initiation of amiodarone: a case report. Mil Med.

[5] Grewal H, Singh A, Thapa S (2018). Am I drowning in blood? Or amio-daroning. Chest.

[6] Martin WJ II, Rosenow EC III (1988). Amiodarone pulmonary toxicity. Recognition and pathogenesis (Part I). Chest.

[7] Wolkove N, Baltzan M (2009). Amiodarone pulmonary toxicity. Can Respir J.

[8] Valle JM, Alvarez D, Antunez J, Valdes L (1995). Bronchiolitis obliterans organizing pneumonia secondary to amiodarone: a rare aetiology. Eur Respir J.

[9] Papiris SA, Triantafillidou C, Kolilekas L, Markoulaki D, Manali ED (2010). Amiodarone: review of pulmonary effects and toxicity. Drug Saf.

[10] Duello KM, Louh IK, Burger CD (2012). 48-Year-old woman with dyspnea, cough, and weight loss. Mayo Clin Proc.

[11] Naranjo CA, Busto U, Sellers EM (1981). A method for estimating the probability of adverse drug reactions. Clin Pharmacol Ther.

[12] Ioachimescu OC, Stoller JK (2008). Diffuse alveolar hemorrhage: diagnosing it and finding the cause. Cleve Clin J Med.

[13] McDonald MG, Au NT, Wittkowsky AK, Rettie AE (2012). Warfarin-amiodarone drug-drug interactions: determination of [I](u)/K(I,u) for amiodarone and its plasma metabolites. Clin Pharmacol Ther.

[14] Kaya B, Yildiz I, Baha RM, Zeytun NE, Yetisgen A (2015). Diffuse alveolar hemorrhage associated with warfarin therapy. Case Rep Med.

